# How to Pay for Memorial Schemes

**Published:** 1919-09-20

**Authors:** 


### HOW TO PAY FOR MEMORIAL SCHEMES.

### A Criticism and a Suggestion.

The complaint that localities are often indifferent to the war memorials which they are expected to establish is not usually made in public, but it is felt. Admitting the feeling, let us see if its root is necessarily as little respectable as it appears, and if there is not a simple remedy for it. English people are quarrelsome rather than clubbable by nature, and that explains why only their voluntary associations work well. The committees which they join under pressure of some kind work less well, and memorial committees are usually recruited under pressure. Then the inert local public has to be interested. How?

The wrong way is seen in a circular issued on behalf of a village church war memorial, which lies before us as we write. The memorial is to take the form of a stone cross in the churchyard and of inscribed brasses in the church. On the latter the name, rank, and regiment of all the parishioners who fell are to be recorded. The circular proceeds:

> All will naturally wish to contribute to the cost of the memorial, but many may be kept back from doing so because they can only afford a small sum; the smallest amount, however, will be gladly received.

What villager will respond to that? It is likely, if anything, to discourage subscriptions, and we venture to suggest that the paragraph should have been written:

> All will naturally wish to contribute to the cost of the memorial, but some may be kept from doing so because they do not realise for how small a sum the names of those whom they love can be thus commemorated. There are expected to be about ——— names. The cost of inscribing these names on brasses is about ———. This means that each name can be recorded for ———. Every family will desire to see a certain name or names recorded. Since the cost per name works out at ———, each family will know exactly the cost of its own share in the complete scheme. Not only will it know th. but the knowledge will enable it to make the memorial personal as well as public by paying for the cost of its own record, which, too, as part of a larger record, will be less costly and more dignified than a number of separate records would be.

Such a plan would be easily understood, and probably popular. The cost of the cross either could be paid for by separate donations, or, if these were insufficient, by supplementing them with a *pro rata* sum, which each family should be invited to add to the money required to pay for the cost of its own share in the brasses. The church in question is that of Uckfield, Sussex, and we mention the place that the rector may consider our suggestion—a suggestion which applies to every hospital and town with similar desires. If the suggestion is studied in connection with the article on "War Memorials for Small Hospitals" published last week in The Hospital, we believe that no memorial scheme of the kind need fail for want of attention to method or lack of beauty in the result.

